# The Impact of Family Visits on Delirium in Patients Admitted to the Intensive Care Unit: A Systematic Review

**DOI:** 10.7759/cureus.94066

**Published:** 2025-10-07

**Authors:** Yogesh Manhas, Sulaiman Al Rahbi, Abhijit Nair

**Affiliations:** 1 Critical Care, Ibra Hospital, Ibra, OMN; 2 Anesthesiology, Ibra Hospital, Ibra, OMN

**Keywords:** family visits, icu delirium, intensive care unit, systematic review and meta analysis, visiting hours

## Abstract

Delirium is an acute disturbance in attention and awareness that affects patients admitted to the ICUs, and its incidence is particularly high in mechanically ventilated patients. This systematic review aimed to evaluate the impact of liberalizing family visiting hours on reducing the incidence of delirium in adult ICU patients. A systematic search was conducted across PubMed, Scopus, and Cumulative Index to Nursing and Allied Health Literature (CINAHL) following Preferred Reporting Items for Systematic reviews and Meta-Analyses (PRISMA) guidelines. This review included randomized controlled trials (RCTs) and observational studies comparing liberal or frequent family visiting strategies versus restrictive visiting policies in adult ICU patients. The primary outcome was the incidence of delirium in the ICU, which was assessed by the confusion assessment method (CAM) or Intensive Care Delirium Screening Checklist (ICDSC). It was prospectively registered with PROSPERO.

The pooled analysis of four studies (512 delirium events among 2291 patients in the flexible group vs. 382 events among 1793 patients in the restricted group) found no statistically significant difference in delirium incidence between both groups (risk ratio (RR): 0.82; 95% confidence interval (CI): 0.60-1.12; p=0.21), with substantial heterogeneity (I²=77%). This systematic review and meta-analysis found no significant reduction in the incidence of ICU delirium with liberal family visiting hours. The limitations of the review include methodological heterogeneity, high risk of bias, and variability in visitation protocols and delirium assessment methods among included studies. We recommend well-designed studies in the future to assess the true impact of flexible visiting polices in the ICU.

## Introduction and background

Delirium is defined by the Diagnostic and Statistical Manual of Mental Disorders, Fifth Edition (DSM-5) as an acute disturbance in attention and awareness [1]. The key features of the condition include disturbance in attention and awareness, developing over a short period and tending to fluctuate, and the presence of neurocognitive deficits, which are not explained by another preexisting, evolving, or established neurocognitive disorder or the presence of coma. Delirium affects 30-50% of patients admitted to ICUs. The reported incidence is even higher, up to 80% among patients receiving mechanical ventilation [2].

The majority of ICUs across the world restrict family visits, based on the perceived notion that such visits interfere with ICU work and may increase the risk of infections among patients. However, current evidence has challenged the notion and supports more flexible, family-centered policies [3-4]. Delirium in critically ill patients is associated with longer ventilation times, increased mortality, and persistent cognitive impairment [5]. Studies have reported an association of increased morbidity and mortality with the occurrence of delirium in ICU patients [5-7].

The primary tool for assessment of delirium in the ICU is the confusion assessment method (CAM), which has a sensitivity and specificity of 94-100% and 90-95% respectively [8]. Intensive Care Delirium Screening Checklist (ICDSC) is another validated screening tool for the diagnosis of delirium in the ICU. Several studies have described the benefits of liberalizing visiting hours and extended family presence at the bedside of ICU patients [9-11]. Allowing family members at the bedside could reduce overall stress and cognitive stimulation, which may help reduce the risk of delirium [12,13]. This systematic review aims to investigate whether liberalizing family visiting hours reduces the incidence of delirium in ICU patients.

## Review

Methods

The Preferred Reporting Items for Systematic Reviews and Meta-Analyses (PRISMA) guidelines were followed in the conduct of this systematic review and meta-analysis. The review was prospectively registered with PROSPERO (https://www.crd.york.ac.uk/PROSPERO), with registration number: CRD42025641063.

Search Strategy

We searched PubMed, Scopus, and Cumulative Index to Nursing and Allied Health Literature (CINAHL) for randomized-controlled trials (RCTs) that compared frequent versus restrictive visiting strategies for family members visiting adult patients admitted to the ICU. The search strategy used with various databases is summarized in Table 1. The primary outcome was the incidence of delirium in both groups. The secondary outcomes were length of ICU stay, duration of visits, and mortality.

**Table 1:** Details of various database searches CINAHL: Cumulative Index to Nursing and Allied Health Literature

Database	Truncation/search strategy
PubMed	((family visit) AND (delirium)) AND (intensive care unit) Filters: from 2010/1/1 - 2025/3/31 Sort by: Publication Date (("familialities"[All Fields] OR "familiality"[All Fields] OR "familially"[All Fields] OR "familials"[All Fields] OR "familie"[All Fields] OR "family"[MeSH Terms] OR "family"[All Fields] OR "familial"[All Fields] OR "families"[All Fields] OR "family s"[All Fields] OR "familys"[All Fields]) AND ("visit"[All Fields] OR "visitation"[All Fields] OR "visitations"[All Fields] OR "visited"[All Fields] OR "visiting"[All Fields] OR "visits"[All Fields]) AND ("delirium"[MeSH Terms] OR "delirium"[All Fields] OR "delirium s"[All Fields] OR "deliriums"[All Fields]) AND ("intensive care units"[MeSH Terms] OR ("intensive"[All Fields] AND "care"[All Fields] AND "units"[All Fields]) OR "intensive care units"[All Fields] OR ("intensive"[All Fields] AND "care"[All Fields] AND "unit"[All Fields]) OR "intensive care unit"[All Fields])) AND (2010/1/1:2025/3/31[pdat]) Translations family: "familialities"[All Fields] OR "familiality"[All Fields] OR "familially"[All Fields] OR "familials"[All Fields] OR "familie"[All Fields] OR "family"[MeSH Terms] OR "family"[All Fields] OR "familial"[All Fields] OR "families"[All Fields] OR "family's"[All Fields] OR "familys"[All Fields] visit: "visit"[All Fields] OR "visitation"[All Fields] OR "visitations"[All Fields] OR "visited"[All Fields] OR "visiting"[All Fields] OR "visits"[All Fields] delirium: "delirium"[MeSH Terms] OR "delirium"[All Fields] OR "delirium's"[All Fields] OR "deliriums"[All Fields] intensive care unit: "intensive care units"[MeSH Terms] OR ("intensive"[All Fields] AND "care"[All Fields] AND "units"[All Fields]) OR "intensive care units"[All Fields] OR ("intensive"[All Fields] AND "care"[All Fields] AND "unit"[All Fields]) OR "intensive care unit"[All Fields]
Scopus	(TITLE-ABS-KEY (family visit) AND TITLE-ABS-KEY (intensive care unit) AND TITLE-ABS-KEY (delirium) ) AND PUBYEAR >2006 AND PUBYEAR <2026
CINAHL	family support AND intensive care unit AND delirium

Eligibility Criteria

The Population, Intervention, Control, Outcome, and Study (PICOS) design was employed to choose relevant studies. In the quantitative analysis, both observational studies and RCTs were included. Systematic reviews, literature reviews, scoping reviews, case reports, series, editorials, conference abstracts, research involving animals, studies without full-text availability, and studies written in languages other than English were excluded. The inclusion criteria comprised RCTs and observational studies involving adult patients admitted to the ICU. The intervention to be studied was allowing frequent visits by family members. The control consisted of allowing restricted visits by family members. The primary outcome to be assessed was the incidence of delirium.

Data Extraction

The initially identified studies were screened by two authors (YM and AN) per the inclusion and exclusion criteria. If the two authors disagreed about the topic of study inclusion, another author (SAR) was asked to settle the disagreement. The following headings were used to summarize the details of each included article once they were finalized: author and year of publication, country, study type, number of participants per intervention arm, description of intervention and control, and primary and secondary outcomes. 

Methodological Assessment

Using the Risk of Bias (ROB2) tool, two authors (YM and AN) independently evaluated the methodological quality of the chosen articles in accordance with the recommendations made by the Cochrane Intervention System Evaluation Manual (Cochrane Handbook for Systematic Reviews of Interventions) [14]. If the two researchers were unable to agree on the methodological evaluation, a third researcher (SAR) assisted in the conclusion. Bias resulting from the randomization process, bias resulting from deviations from the intended intervention, bias resulting from missing outcome data, bias in outcome measurement, and bias in the selection of the reported result were all included in the methodological assessment (ROB2). The observational studies were assessed for risk of bias using the ROBINS-E tool. A total of seven parameters were considered for risk of bias assessment: confounding, measurement of the exposure, selection of participants into the study, post-exposure interventions, missing data, measurement of outcome, and selection of reported results [15].

Quantitative Meta-Analysis

Review Manager software (RevMan 5.4) was used by the Cochrane Collaboration to conduct meta-analysis [16]. For binary variables, the 95 percent confidence interval (CI) and the risk ratio (RR) were employed. For continuous variables, the 95% CI and mean difference (MD) were used. A comparison of similar groups using a forest plot analysis yielded the effect estimates. A p-value was considered statistically significant if it was less than 0.05. We used the χ2 and I^2^ to measure clinical heterogeneity in the included studies [17]. If the study results demonstrated low heterogeneity (p>0.10, I^2^<50%), a fixed-effects model was employed. The random-effects model was applied in every other instance [18]. To evaluate its impact on the overall effect size, we conducted a leave-one-out sensitivity analysis by eliminating the trial with the highest weight.

Results

**Figure 1:**
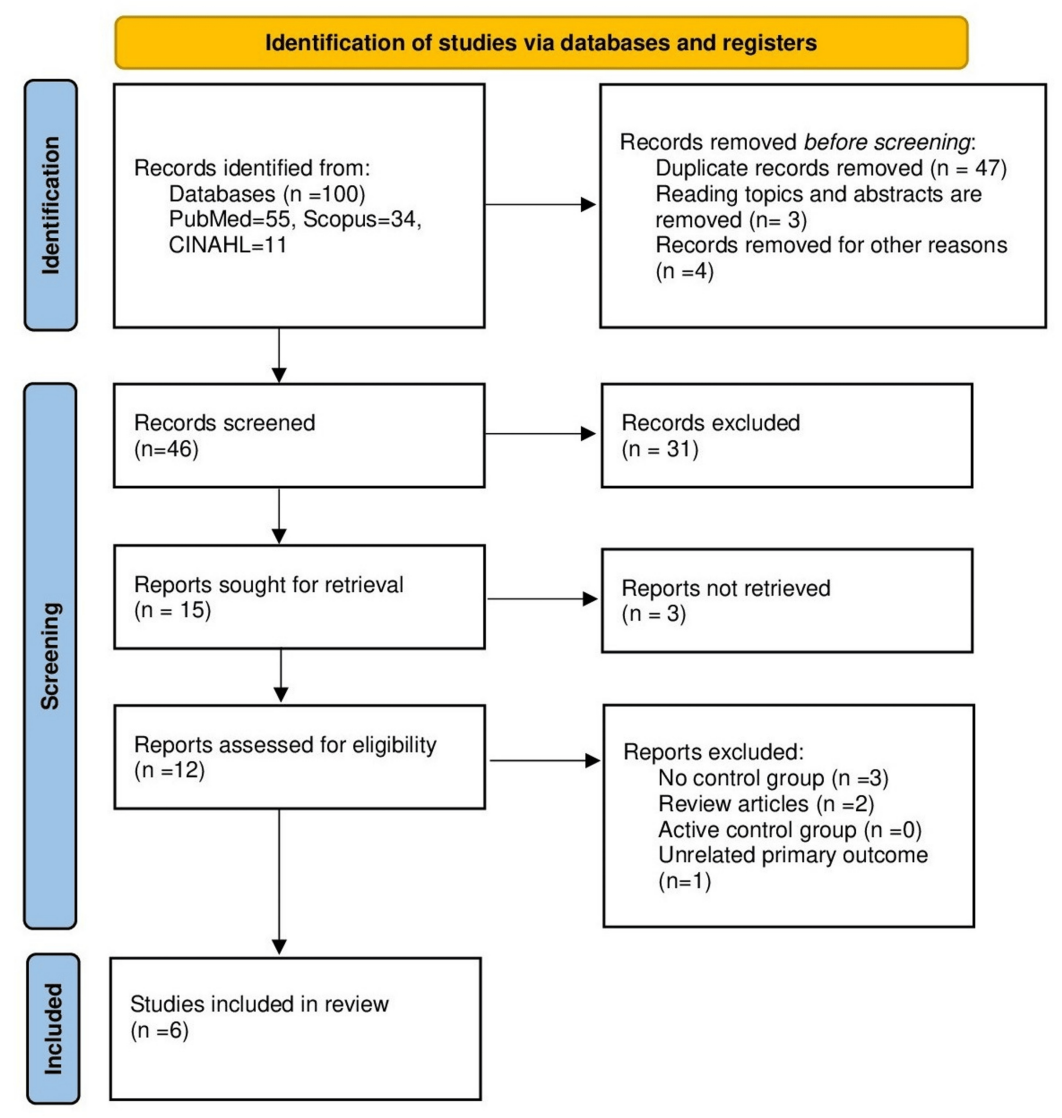
PRISMA flow diagram depicting the selection of studies PRISMA: Preferred Reporting Items for Systematic reviews and Meta-Analyses

We identified 100 articles based on the inclusion criteria mentioned above. After removing duplicates and excluding articles that were not relevant, 46 titles were screened, of which 31 were excluded. From the remaining articles, three articles were not retrieved as they were not relevant. Of the remaining 12 articles, six were excluded (three with no control group, two review articles, and one with unrelated primary outcomes). Finally, six studies were selected for a qualitative systematic review and a quantitative meta-analysis [13,19-23] (Figure 1). Study characteristics and outcome details are summarized in Tables 2-3.

**Table 2:** Summary of the included studies ICU: intensive care unit; CAM: confusion assessment method; ICDSC: Intensive Care Delirium Screening Checklist; APACHE: Acute Physiology and Chronic Health Evaluation

Author(s) and year	Country	Study period	Number of patients	Intervention	Control	Reported outcomes (primary)	Reported outcomes (secondary)
Rosa et al., 2017 [13]	Brazil	May 2015 to November 2015	Intervention: 145, control: 141	Extended visitation model: 12 hours/day	Restricted visitation model: 4.5 hours/day	Cumulative incidence of delirium using CAM-ICU	Duration of delirium/coma. ICU-acquired bloodstream infection, pneumonia, and urinary tract infection; all-cause ICU mortality and length of ICU stay
Eghbali-Babadi et al., 2017 [19]	Iran	2013	Intervention: 34, control: 34	The day after surgery, a family member who received education about communication methods and prevention of delirium was allowed in the morning shift	Routine care	Incidence of delirium measured by CAM-ICU	None
Kim et al., 2022 [20]	South Korea	January 2019 to May 2021	Retrospective observational study: 2196 patients. 1632 enrolled before the ICU visit ban, and 564 enrolled after the ICU visit ban	Restricted visiting by family members	No visiting by family members	Incidence of Delirium measured by CAM-ICU	To estimate the subtypes of delirium, compare other variables like APACHE, sedation, and mechanical ventilation
Mohsen et al., 2022 [21]	Canada	January 2014 to December 2018	Retrospective cohort of 25,537 patients >18 years admitted at least once in the ICU. Physical family presence=23,121. Family involvement through a telephone call=591. No visit=1825	Family presence (physically or telephonically)	No family visit	Prevalence of delirium measured using ICDSC is defined as an ICDSC score greater than or equal to 4	Duration of delirium
Rosa et al., 2019 [22]	Brazil	April 2017 to June 2018	Intervention: 837, control: 848	Flexible visiting hours up to 12 hours/day	Restricted visiting hours: 1.5 hours/day	Primary outcome - Incidence of delirium during ICU stay using the CAM ICU scoring system	ICU-acquired infection, anxiety, and depression among family members using the HADS score, and burnout among ICU staff using the Maslach Burnout Inventory
Westphal et al., 2018 [23]	Brazil	March 2015 to February 2017	Intervention: 268, control: 248	Extended visitation up to 24hours/day	Restricted visitation up to 6 hours/day	Incidence of Delirium measured by ICDSC	Healthcare-associated infections, length of stay

**Table 3:** Details of various outcomes in the included studies ICU: intensive care unit; RR: risk ratio; OR: odds ratio; CI: confidence interval

S. no.	Study	Country	Groups	No of patients	Outcomes
1	Rosa et al., 2019 [22]	Brazil	Flexible visiting hours up to 12 hours/day	837	Primary outcome: delirium in 157 out of 831 (18.9%). Other outcomes: anxiety prevalence in family: 13.4%, burnout among staff: 22%
			Restricted visiting hours: 1.5 hours/day	848	Primary outcome: delirium in 170 out of 845 (20.1%). Other outcomes: anxiety prevalence in family: 28.2%, burnout among staff: 24.8%
2	Mohsen et al., 2022 [21]	Canada	Physical family presence	23,121	Primary outcome: delirium duration and family presence: –1.87 (–2.01 to –1.81); p<0.001 (n=13,984). Other outcomes: hospital LOS (median): 13 (6–27). ICU LOS (median): 4.5 (2.6–8.6). Death in % (ICU): 2,076 (9.0). Death in % (hospital): 3479 (15.1)
			Family involvement through a telephone call	591	Primary outcome: delirium duration and family presence: –1.41 (–1.52 to –1.31); p<0.001 (n=289). Other outcomes: hospital LOS (median): 10 (5–20.5). ICU LOS (median): 2.7 (1.9–4.1). Death in % (ICU): 11 (1.9). Death in % (hospital): 32 (5.4)
			No visit	1825	Other outcomes: hospital LOS (median): 9 (4–17). ICU LOS (median): 2.1 (1.6–3.4). Death in % (ICU): 20 (1.1). Death in % (hospital): 68 (3.7)
3	Kimet al., 2022 [20]	South Korea	Patient enrolled before the ICU visit ban	1632	Incidence of delirium: 323 out of 1047 patients (30.9%) (p=0.162). Delirium subtype: hyperactive: 27.7% (p=0.002). Anxiety: 52.22% (standard deviation: 6.50), p=0.009
			Patient enrolled after the ICU visit ban	564	Incidence of delirium: 153 out of 559 patients (27.4%) (p=0.162). Delirium subtype: hyperactive: 35.3% (p=0.002). Anxiety: 53.46% (standard deviation: 4.58), p=0.009
4	Eghbali-Babadi et al., 2017 [19]	Iran	Morning after surgery (day 2) visit by the family members	34	Incidence of delirium on the day after the surgery: 11.76% (4/34). Incidence of delirium on 3^rd^ day: 8.83%
			Morning after surgery (day 2), no visit by family member	34	Incidence of delirium on the day after the surgery: 23.53% (8/34). Incidence of delirium on 3^rd^ day: 20.58%
5	Rosa RG et al., 2017 [13]	Brazil	Extended visit: 12 hours/day	145	Primary outcome: cumulative incidence of delirium: 9.6%. Adjusted RR: -0.50, CI: (0.26- 0.95), p=0.03. Other outcomes: median duration of delirium: 1.5 days (p=0.03). Rate of ICU-acquired infection: no difference. ICU mortality: no difference
			Restricted visit: 4.5 hours/day	141	Primary outcome: cumulative incidence of delirium: 20.50%. Adjusted RR: 0.50, CI: (0.26- 0.95), p=0.03. Other outcomes: median duration of delirium: 3.0 days (p=0.03). Rate of ICU-acquired infection: no difference. ICU mortality: no difference
6	Westphal et al., 2018 [23]	Brazil	Extended visit: 24 hours /day	268	Incidence of delirium: 6.7%. Incidence density of delirium as measured by delirium incidence per 1000 patient days: 15.9, p<0.001
			Restricted visit: 6 hours /day	248	Incidence of delirium: 12.1%. Incidence density of delirium as measured by delirium incidence per 1000 patient days: 29.4, p<0.001

Qualitative Systematic Review

**Figure 2:**
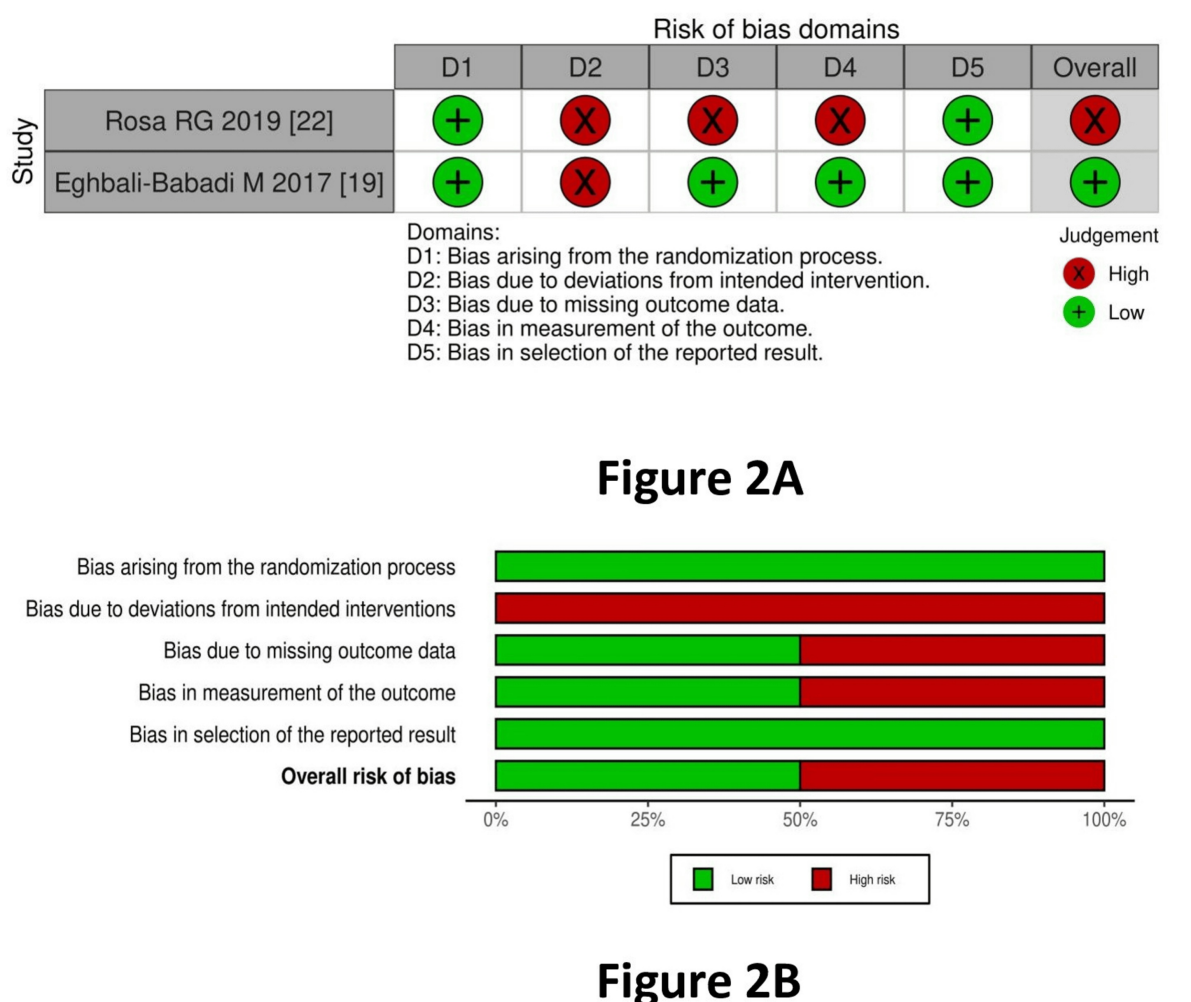
Risk of bias for RCTs A: traffic light plot for RCTs; B: summary plot for RCTs RCTs: randomized-controlled trials

RoB assessment: The risk of bias within the trials for RCTs (two studies) is depicted in Figure 2A (traffic light plot) and Figure 2B (summary plot). This was performed using the RoB2 tool. The bias from the randomization process was low in two studies [13,19]. Bias due to deviations from intended interventions (allocation concealment) was high in both studies [13,19]. Bias arising from missing outcome data was low in one study [19] and high in one study [13]. Bias in the outcome measurement was low in one study [19] and high in one study [18]. Bias arising from the selection of reported results was low in both studies [13,19].

**Figure 3:**
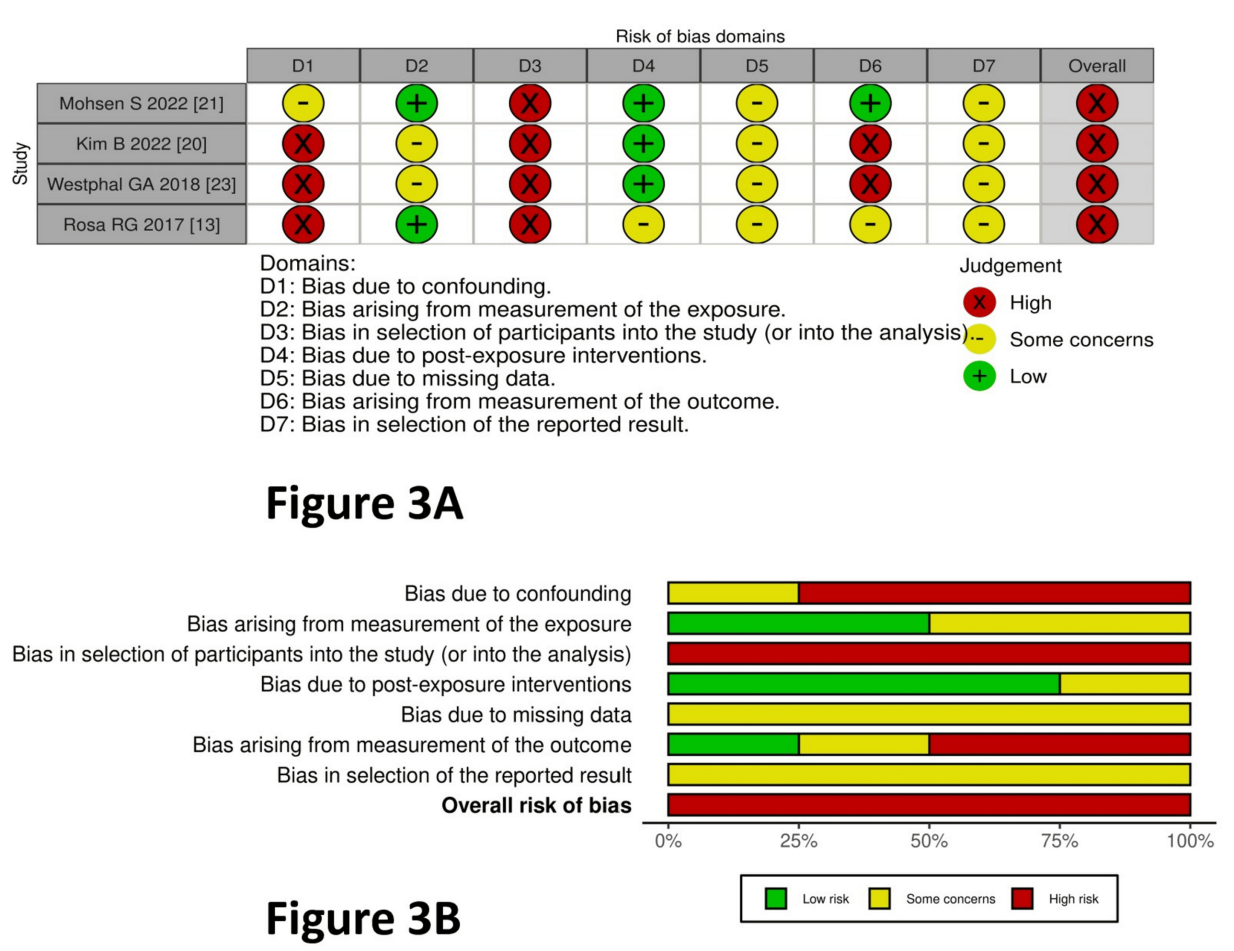
Risk of bias for observational studies A: traffic light plot for observational studies; B: summary plot for observational studies

The risk of bias within the trials for non-RCTs/observational studies (four studies) is depicted in Figure 3A (traffic light plot) and Figure 3B (summary plot). This was performed using the ROBINS-E tool. The bias due to confounding and selection of participants in the study was high in three studies [20,22,23], and there were some concerns in one study [21]. Bias from the measurement of exposure was low in two studies, [21,23] and there were some concerns in two studies [21,22]. Bias in the selection of participants was high in all four studies [20-23]. Bias due to post-exposure interventions was low in three studies [20-22], and there were some concerns in one study [23]. There were some concerns about the bias due to missing data in all four studies [20-23]. Bias arising from measurement of the outcome was high in two studies [20,22], low in one study [22], and there were some concerns in one study [23]. There were some concerns in all four studies regarding bias due to the selection of reported results [20-23]. The overall bias was high in the included observational studies.

Quantitative Meta-Analysis

**Figure 4:**
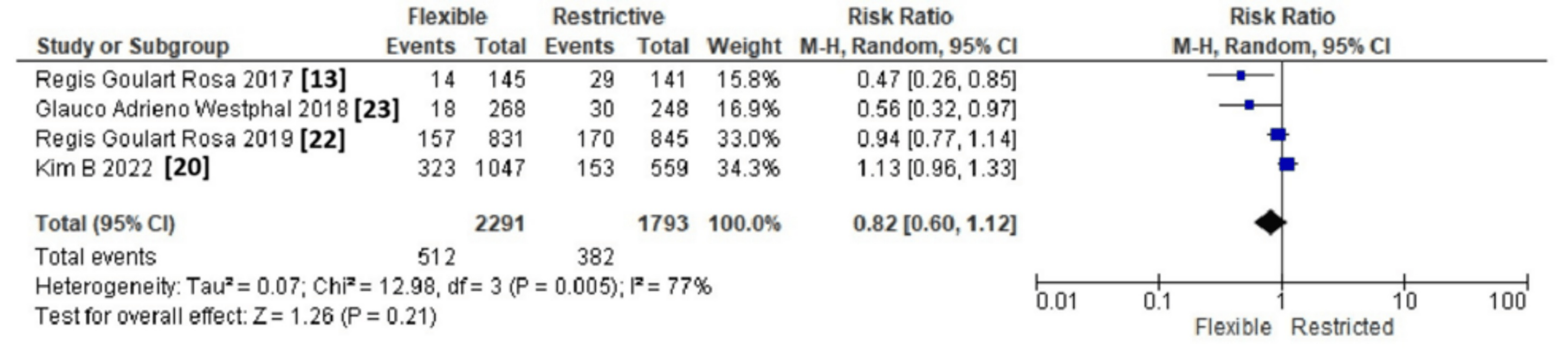
Forest plot showing comparison of incidence of delirium between flexible versus restrictive family visits CI: confidence interval

Of the six included studies, four reported incidence of delirium as an outcome (512 events out of 2291 patients in the flexible group vs. 382 events out of 1793 patients in the restricted visit group) [13,20,22,23]. A pooled analysis revealed no significant difference in the incidence of delirium between the two groups (risk ratio: 0.82; 95% CI: 0.60, 1.12, p=0.21). The overall heterogeneity was high using a random effect model (I^2^=77%) (Figure 4).

In a predefined sensitivity analysis, we excluded the trial by Kim B et al. [20], which contributed 46.5% of the total weight. Without this study, the pooled odds ratio shifted to 0.97 (95% CI: 0.41-1.08; p=0.10; I²=72%), indicating statistical significance, but with still considerable heterogeneity. The lack of statistical significance after removal indicates that no single study disproportionately influenced the overall estimate.

The other outcomes, like anxiety in family members, duration of delirium, length of stay, and mortality, were inconsistently reported in the studies. Therefore, a pooled analysis was not performed. Publication bias was not determined because there were fewer than 10 studies that met the inclusion criteria.

Discussion

Summary of Results

This systematic review investigated the impact of flexible family visiting hours on patients admitted to the ICU versus restricted visiting hours on the incidence of delirium. A qualitative systematic review revealed a high risk of bias in both RCTs and non-RCTs included in the final analysis. A pooled analysis involving four studies showed a comparable incidence of delirium in both groups. This is probably the first review to investigate delirium as an outcome of different approaches to family visiting hours. However, the limitations include including both RCTs and non-RCTs, and significant clinical (different types of patients, medical and surgical) and statistical heterogeneity.

One of the humane approaches to ICU care involves liberalizing visit hours and allowing family to be at the bedside of the patient for longer than the usual restricted visit policies. Family engagement remains one of the important components of the delirium prevention bundle in critically ill patients [24,25]. Several studies have reported potential benefits of unrestricted ICU visit hours for the families without affecting ICU work or an increase in ICU-acquired infections [26-29]. Delirium is a common occurrence in ICU patients, especially those who are mechanically ventilated, and is associated not only with increased morbidity and mortality but also with long-term cognitive impairment [5-7]. Studies have reported a decrease in the incidence of ICU delirium with unrestricted visit hours and active participation of the family in ICU care [13,22,30].

Vitorino et al. published a systematic review without meta-analysis investigating the effectiveness of family participation interventions for the prevention of delirium in patients admitted to the ICU [31]. They analyzed studies that not only included physical involvement of family members, but also involvement with voice messages or phone calls, which we did not include because it does not qualify as satisfactory family member involvement. The authors did mention that the included studies lacked clarity and that the objectives were not clear. Our systematic review included six studies: two RCTs, two before-and-after studies, and two observational studies. Though the individual studies, four out of six, did show decreased incidence of delirium with unrestricted visit hours, a pooled analysis did not show a significant difference in delirium between restricted and unrestricted visit hours.

It is important to note the drawbacks of our meta-analysis, which perhaps make the results debatable. First, there was heterogeneity in the methodology of the included studies. The population sample was heterogeneous in terms of the type of patients and the sample size of the included studies. Different tools were used in the assessment of delirium. In addition, the duration of the visit in the unrestricted group of different studies was variable. The large, randomized trial by Rosa et al. involving 36 adult ICUs in Brazil did not report a reduction in the incidence of delirium with unrestricted family visits [13]. However, it is pertinent to note that the mean duration of family visits in the unrestricted visit group was only 4.8 hours. Though it was significant when compared to 1.4 hours in the restricted group, it may not be sufficient to have a beneficial effect on delirium. Perhaps it is not just the physical presence of the family but the quality of engagement with the patient that matters, which needs to be explored in future studies. Moreover, the study by Rosa et al. excluded patients who had difficulty communicating and those who were in a coma for more than 96 hours. Furthermore, there are difficulties in diagnosing and assessing delirium, which can give an inaccurate estimate of its incidence [32]. Due to the multifactorial nature of delirium, it is challenging to assess the effect of a single intervention on delirium [33,34].

## Conclusions

This systematic review and meta-analysis evaluated the impact of flexible family visiting policies on the incidence of delirium in adult ICU patients. We did not find a decrease in the incidence of delirium in ICU patients with the implementation of an unrestricted visit hours policy. The pooled analysis did not show a significant decrease in delirium with liberalized visiting hours, despite the fact that individual studies demonstrated probable advantages. Our results should be interpreted with caution owing to substantial variations in study designs, patient populations, visitation procedures, and delirium assessment methods. Furthermore, there were different levels of bias risk associated with the included observational studies and RCTs. However, family presence at the patient's bedside remains a crucial part of patient-centered care and might provide benefits beyond delirium prevention, such as improved family satisfaction, communication, and emotional support. To better understand the significance of flexible family visitation policies in the ICU, meticulously planned multicenter trials with standardized visitation protocols and delirium assessment methods are required.
